# Supplementary material for: Acetylcholine esterase inhibitory activity of green synthesized nanosilver by naphthopyrones isolated from marine-derived Aspergillus niger
Source: PLoS One. 2021 Sep 10;16(9):e0257071. doi: 10.1371/journal.pone.0257071 (PMC8432876; doi:10.1371/journal.pone.0257071)
Supplement: S1 File — (PDF) [file pone.0257071.s001.pdf]

Supplementary material

Acetylcholine esterase inhibitory activity of green synthesized nanosilver by naphthopyrones isolated from marine-derived Aspergillus Niger

Ghada Mahmoud Abdelwahab[1,2,*], Amira Mira[1], Yuan-Bin Cheng[4], Tarek A Abdelaziz[3,†] , Mohamed Farid I. Lahloub[1], Ashraf Taha Khalil[1]

[1] Department of Pharmacognosy, Mansoura University, Mansoura, Egypt

[2] Department of Pharmacognosy, Horus University, New Damietta, Egypt

[3] Marine invertebrates, National Institute of Oceanography and Fisheries, Red Sea Branch, Hurghada, Egypt

[4] Department of Marine Biotechnology and Resources, National Sun Yat-sen University, Kaohsiung Taiwan

* Corresponding author

E-mail: ghabdelwahab@horus.edu.eg (GMA)

Editor in Chief: Emily Chenette,

Table of contents

S1 Fig.	^{1}H-NMR spectrum (400 MHz, $CDCl_3$) of compound 1
S2 Fig.	APT spectrum (100 MHz $CDCl_3$) of compound 1.
S3 Fig.	ESI-Mass spectrum (negative ion mode) of compound 1.
S4 Fig.	^{1}H-NMR spectrum (400 MHz, $CDCl_3$) of compound 2.
S5 Fig.	ESI-Mass spectrum (negative ion mode) of compound 2.
S6 Fig.	^{1}H-NMR spectrum (400 MHz, $CDCl_3$) of compound 3.
S7 Fig.	^{13}C NMR spectrum (100 MHz, $CDCl_3$) of compound 3.
S8 Fig.	ESI-Mass spectrum (positive ion mode) of compound 3.
S9 Fig.	^{1}H-NMR spectrum (400 MHz, $CDCl_3$) of compound 4.
S10 Fig.	^{13}C NMR spectrum (100 MHz, $CDCl_3$) of compound 4.
S11 Fig.	ESI-Mass spectrum (positive ion mode) of compound 4.
S12 Fig.	^{1}H-NMR spectrum (400 MHz, $CDCl_3$) of compound 5.
S13 Fig.	^{13}C NMR spectrum (100 MHz, $CDCl_3$) of compound 5.
S14 Fig.	ESI-Mass spectrum (positive ion mode) of compound 5.
S15 Fig.	^{1}H-NMR spectrum (400 MHz, $CDCl_3$) of compound 6.
S16 Fig.	ESI-Mass spectrum (positive ion mode) of compound 6.
S17 Fig.	^{1}H-NMR spectrum (400 MHz, $CDCl_3$) of compound 7.
S18 Fig.	APT spectrum (100 MHz, $CDCl_3$) of compound 7.
S19 Fig.	ESI-Mass spectrum (negative ion mode) of compound 7.
S20 Fig.	^{1}H-NMR spectrum (400 MHz, $CDCl_3$) of compound 3(additional to Figure S10).
S21 Fig.	^{1}H-NMR spectrum (400 MHz, $CDCl_3$) of compound 4(additional to Figure S13).
S1 Table.	UV absorbance of compounds 3, 4 and 7 upon addition of $AgNO_3$ solution **after 1 h. and after 48 h.**

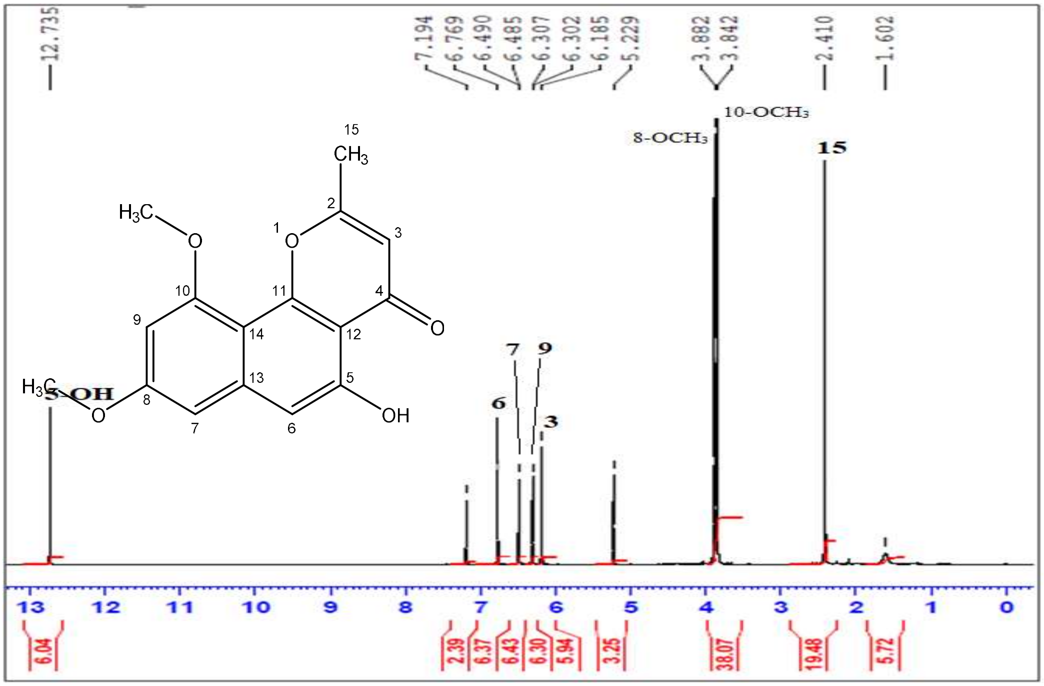

Figure S1 : ^{1}H-NMR spectrum (400 MHz, CD_3OD) of compound 1

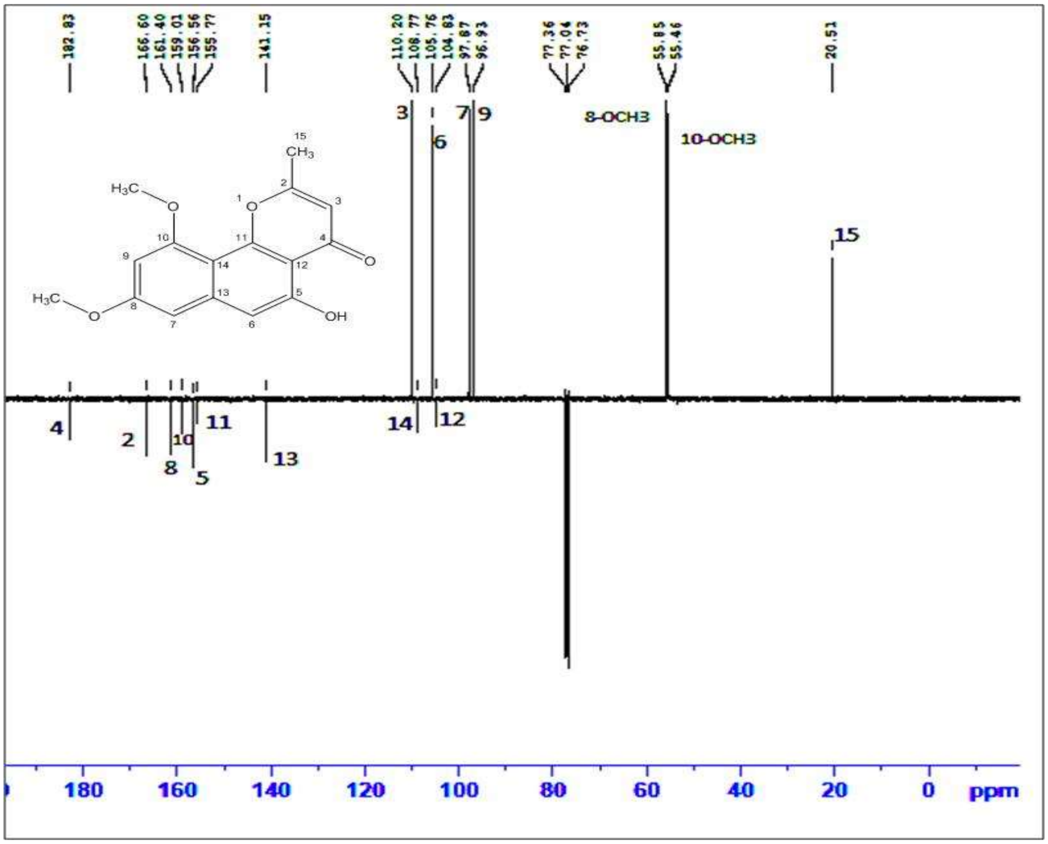

Figure S2: APT spectrum (100 MHz, CD_3OD) of compound 1.

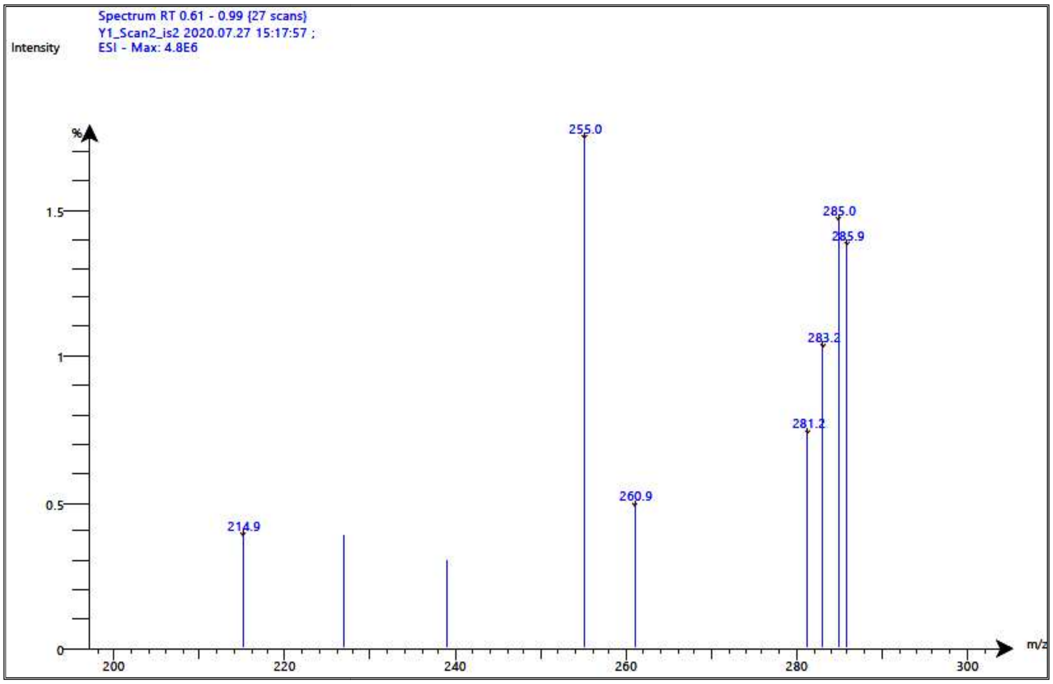

Figure S3 : ESI-Mass spectrum (negative ion mode) of compound 1.

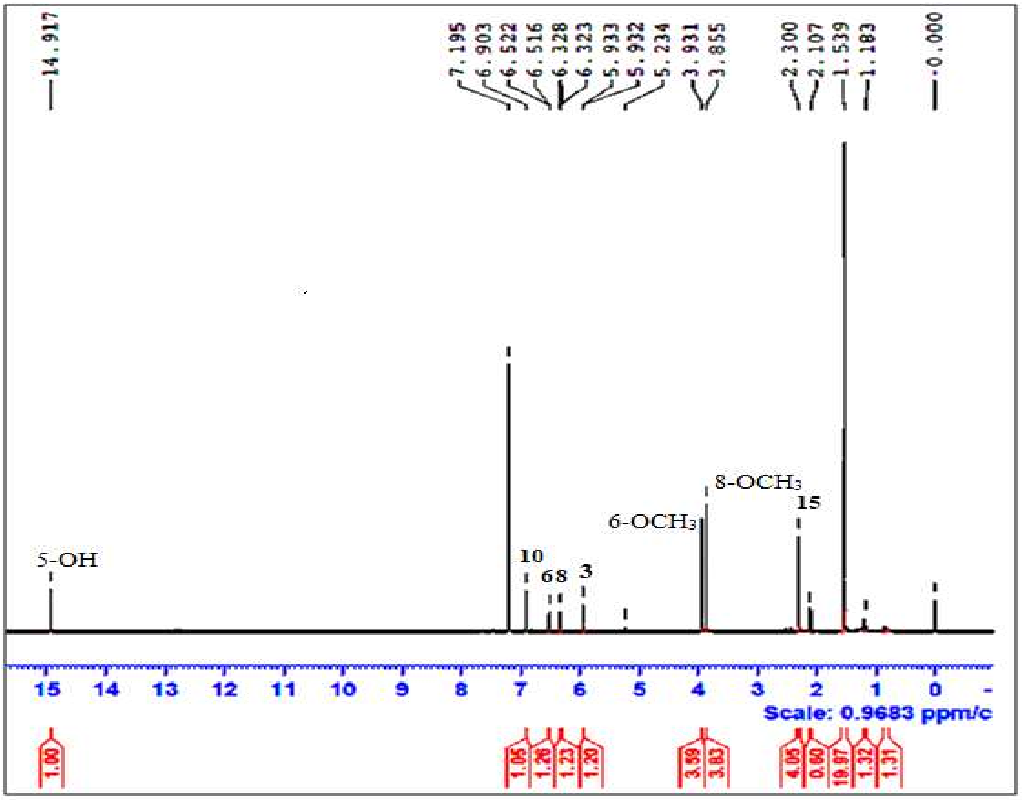

Figure S4 : ^{1}H-NMR spectrum (400 MHz, $CDCl_3$) of compound 2.

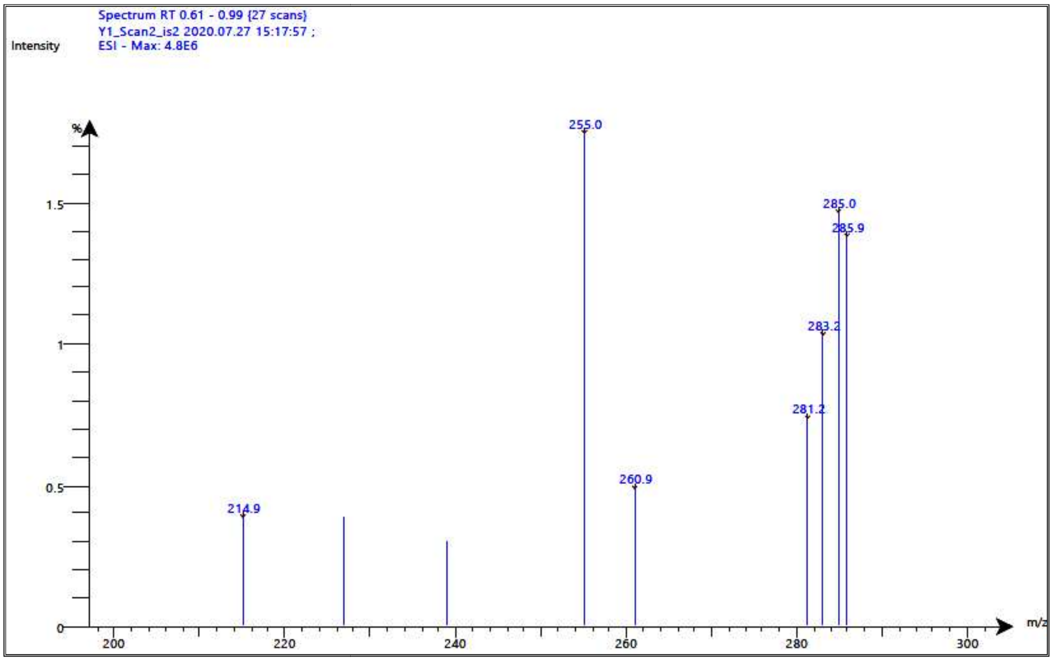

Figure S5 : ESI-Mass spectrum (negative ion mode) of compound 2.

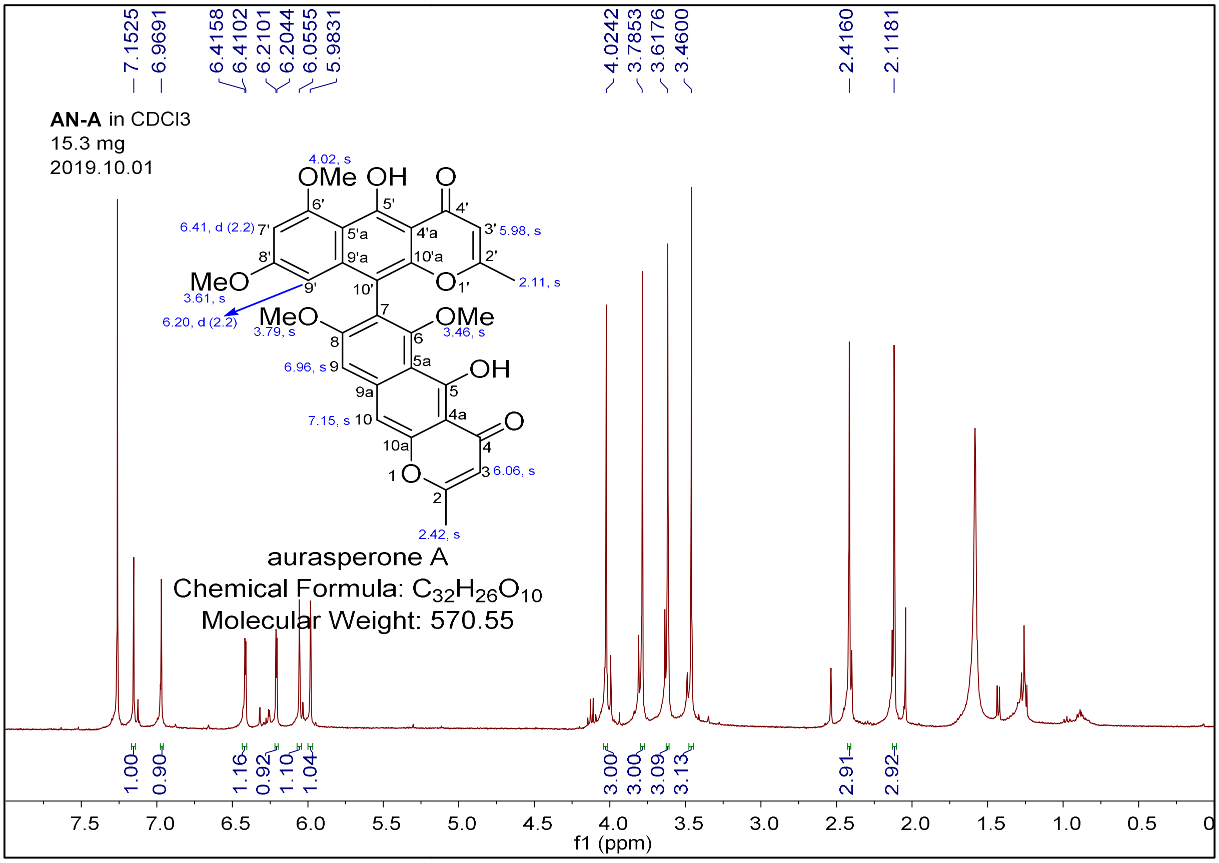

Figure S6 : ^{1}H-NMR spectrum (400 MHz, $CDCl_3$) of compound 3.

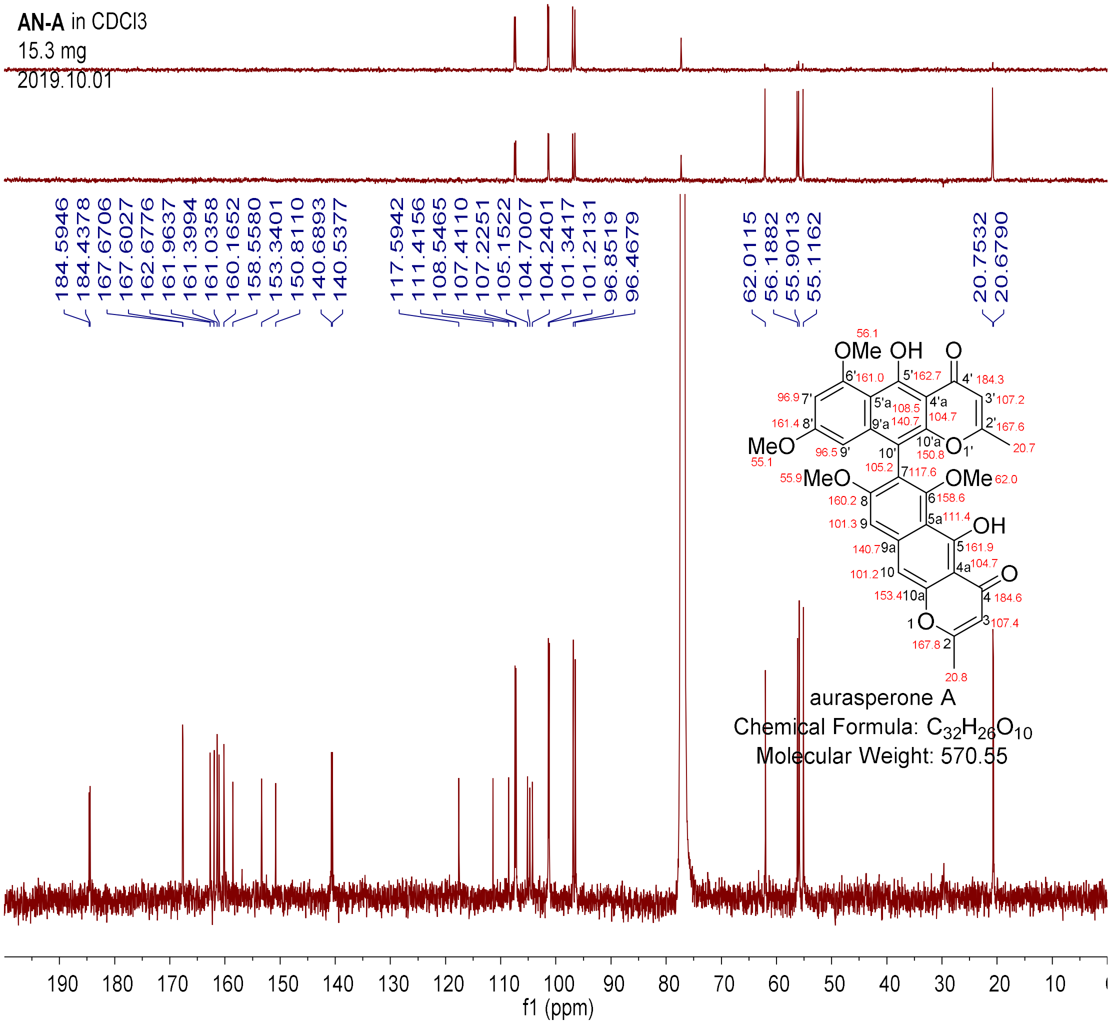

Figure S7 : ^{13}C NMR spectrum (100 MHz, $CDCl_3$) of compound 3.

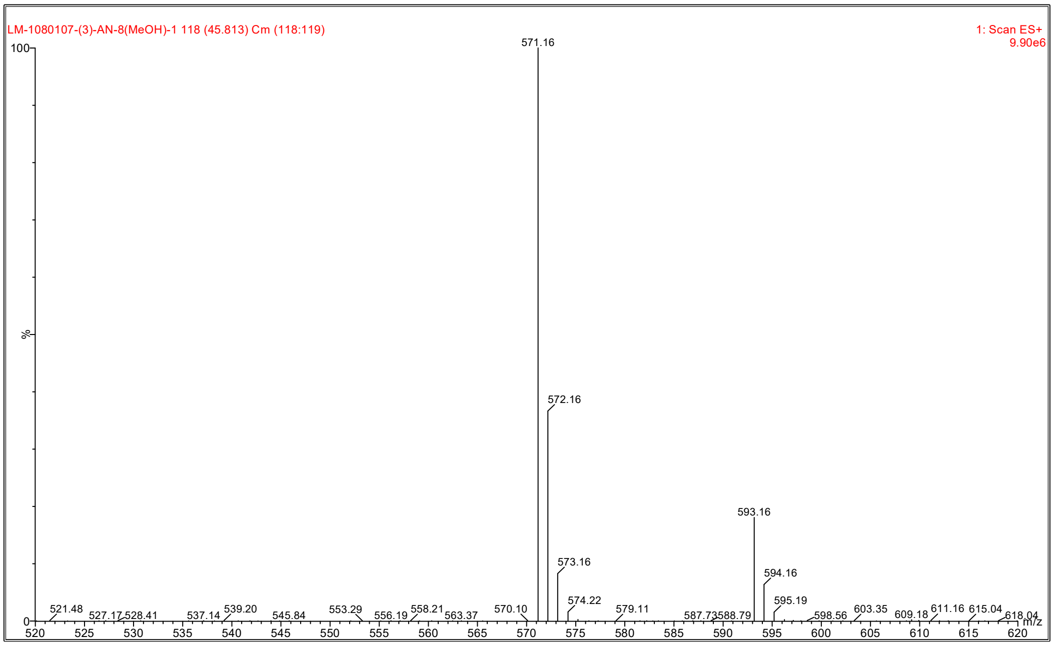

Figure S8 : ESI-Mass spectrum (positive ion mode) of compound 3.

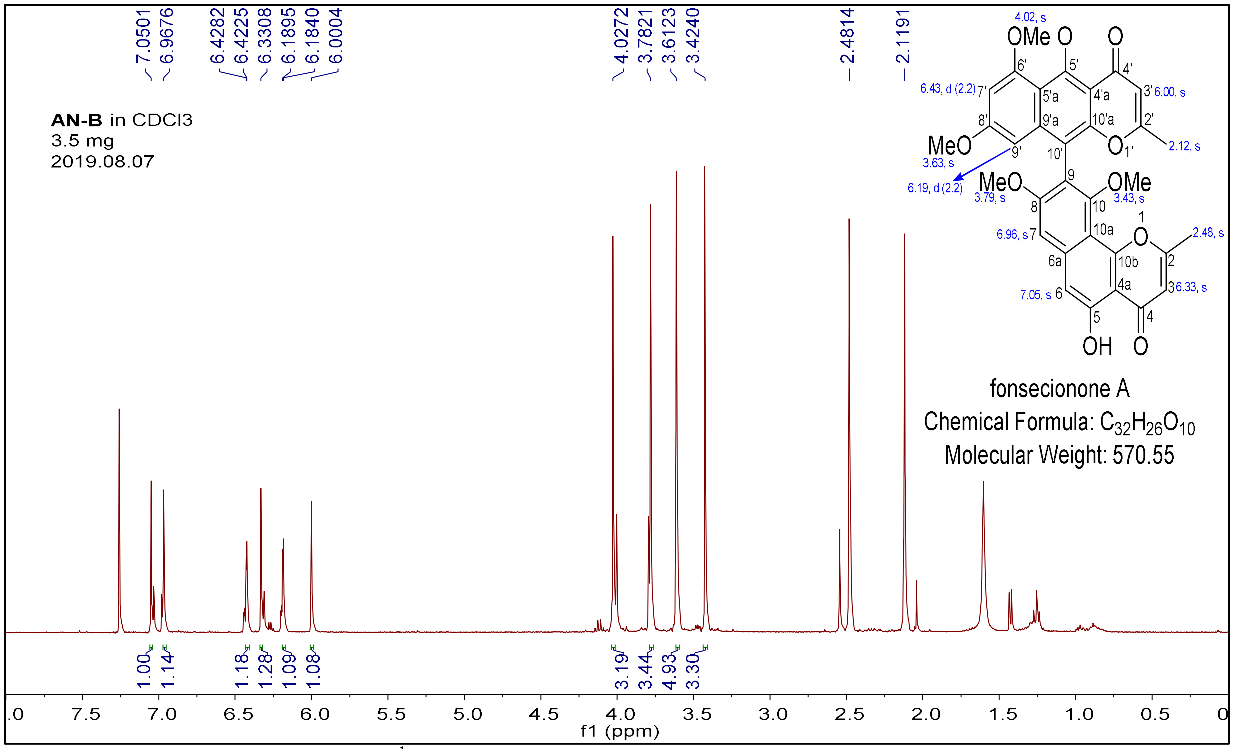

Figure S9 : ^{1}H-NMR spectrum (400 MHz, $CDCl_3$) of compound 4.

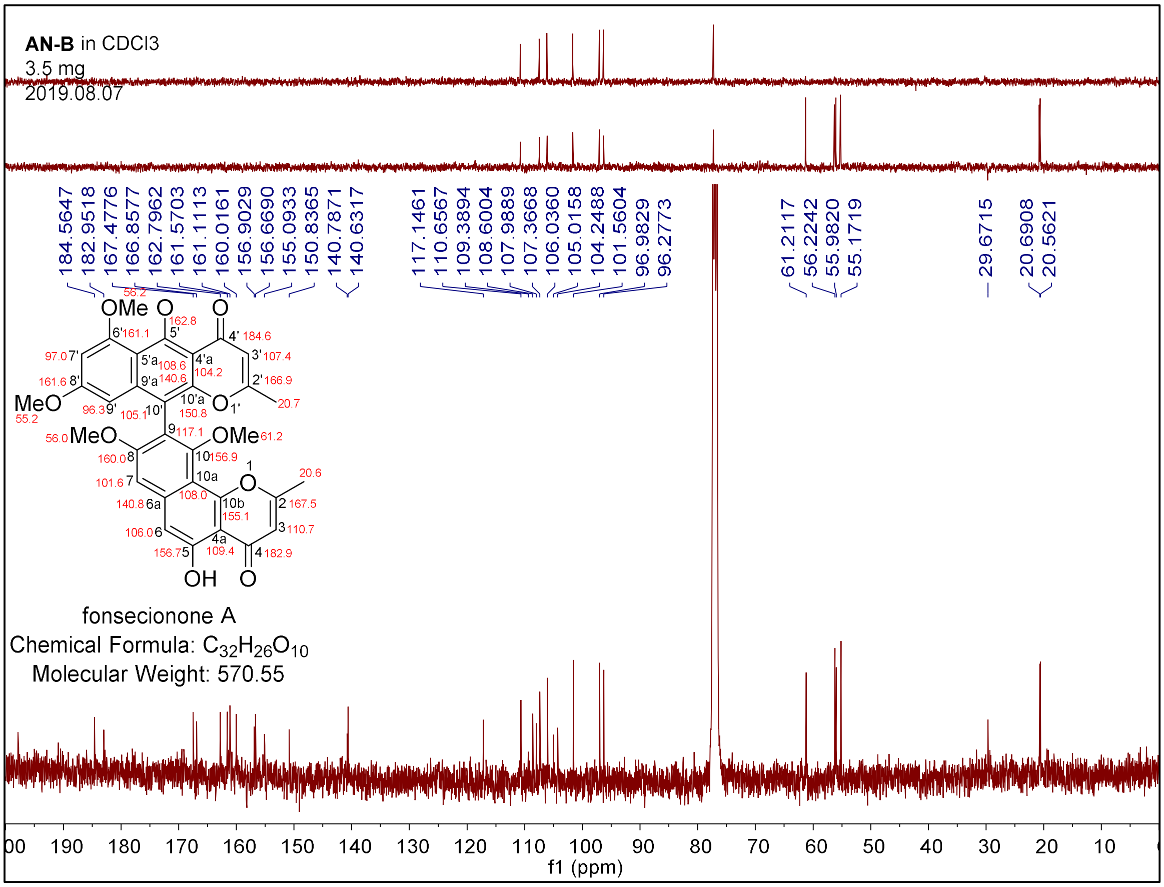

Figure S10 : ^{13}C NMR spectrum (100 MHz, $CDCl_3$) of compound 4.

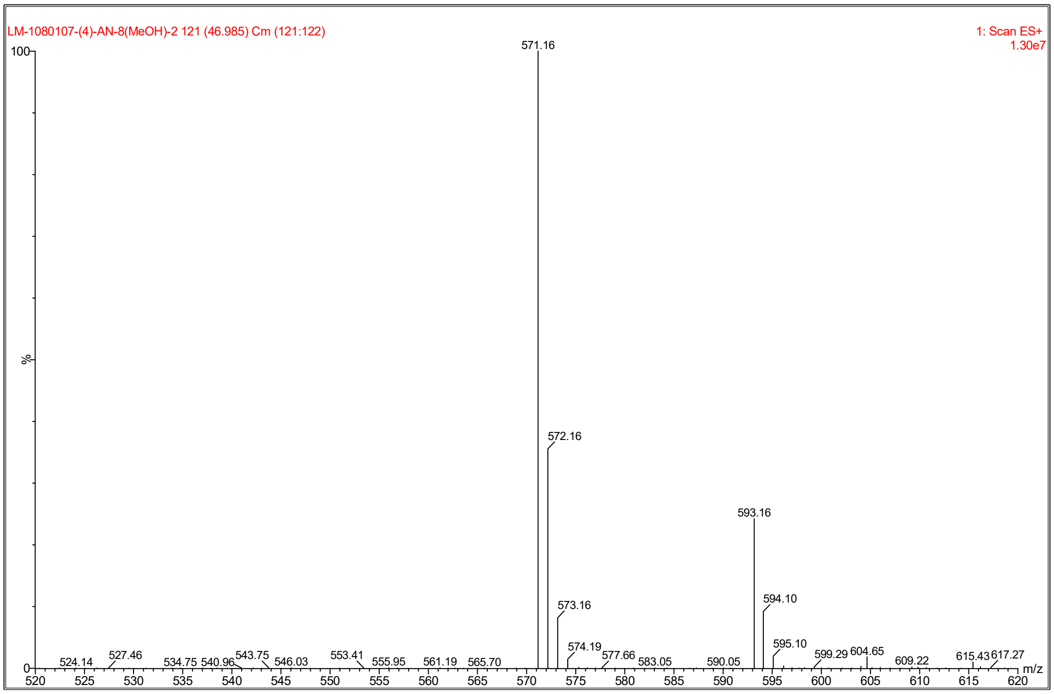

Figure S11 : ESI-Mass spectrum (positive ion mode) of compound 4.

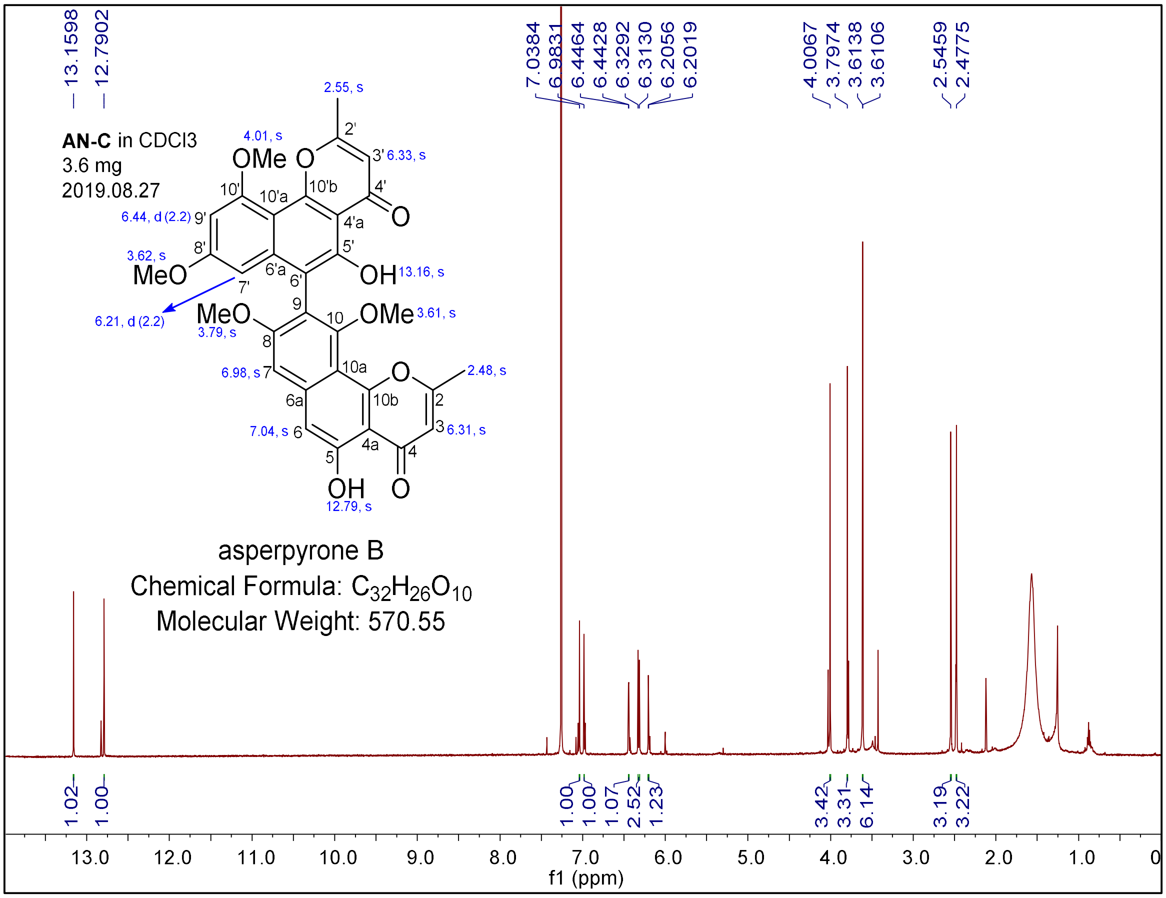

Figure S12 : ^{1}H-NMR spectrum (400 MHz, $CDCl_3$) of compound 5.

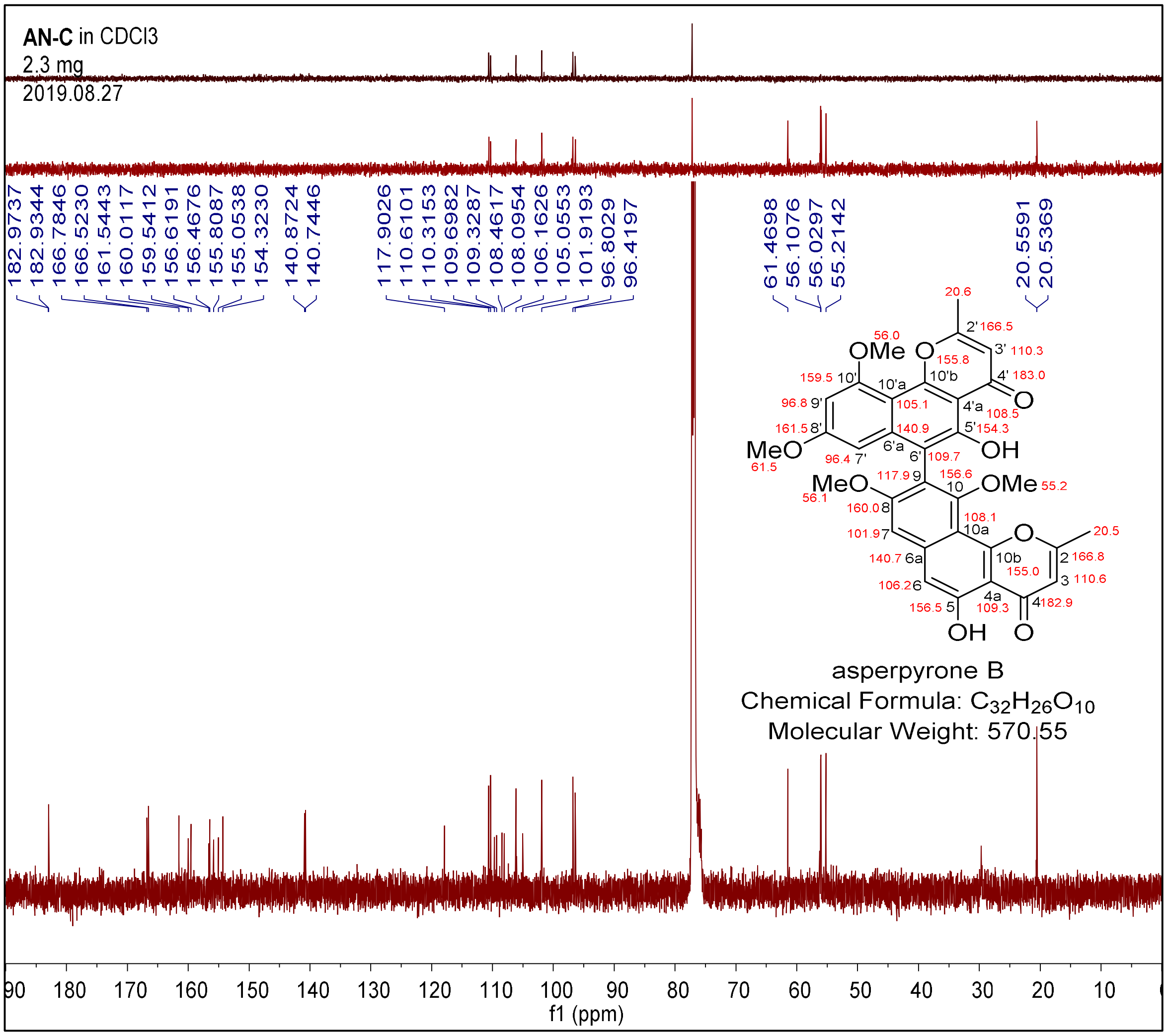

Figure S13 : ^{13}C NMR spectrum (100 MHz, $CDCl_3$) of compound 5.

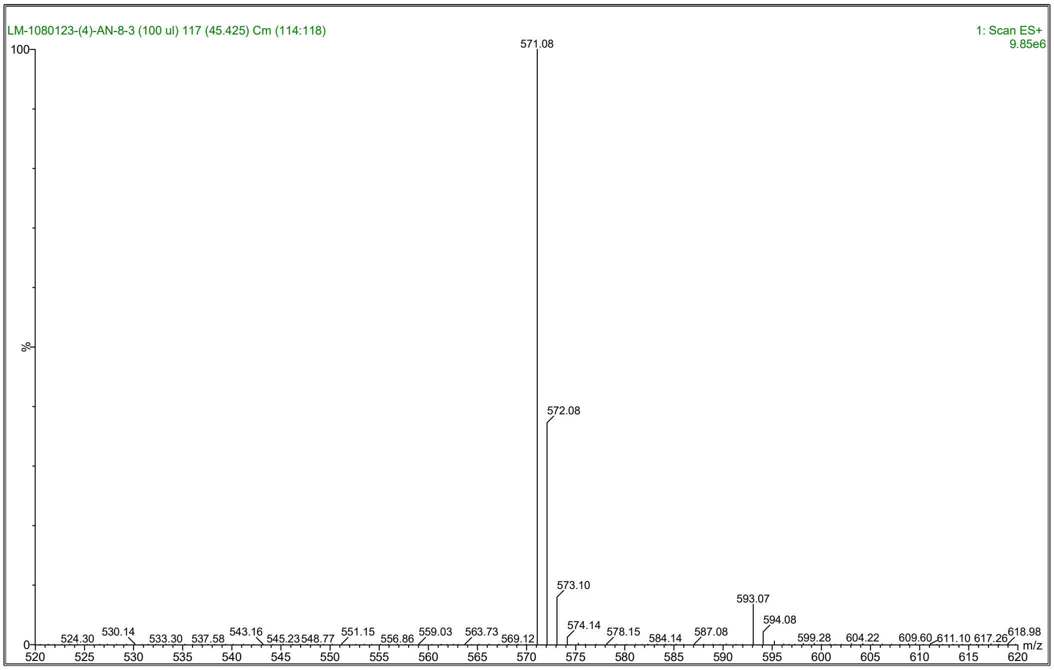

Figure S14 : ESI-Mass spectrum (positive ion mode) of compound 5.

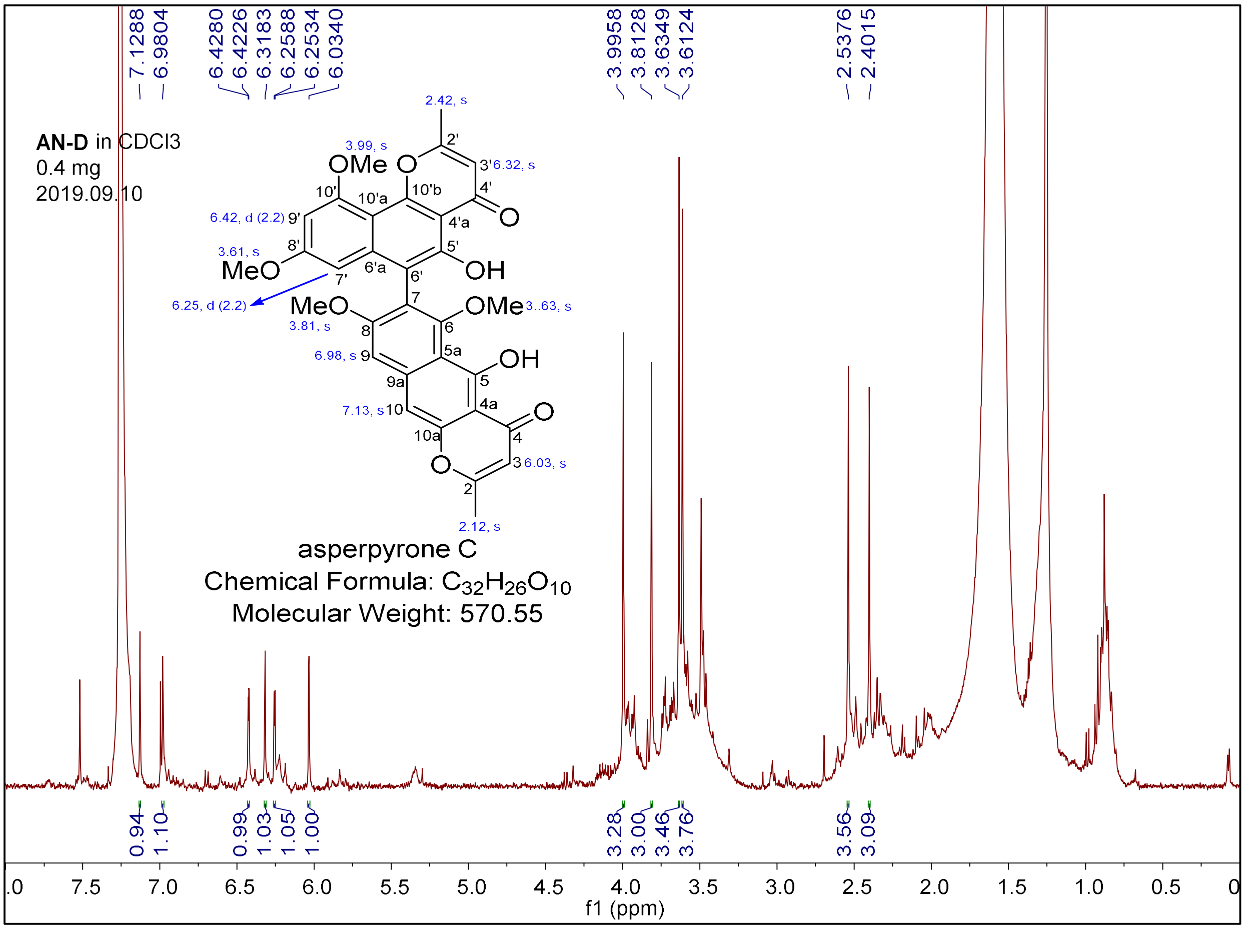

Figure S15 : ^{1}H-NMR spectrum (400 MHz, $CDCl_3$) of compound 6.

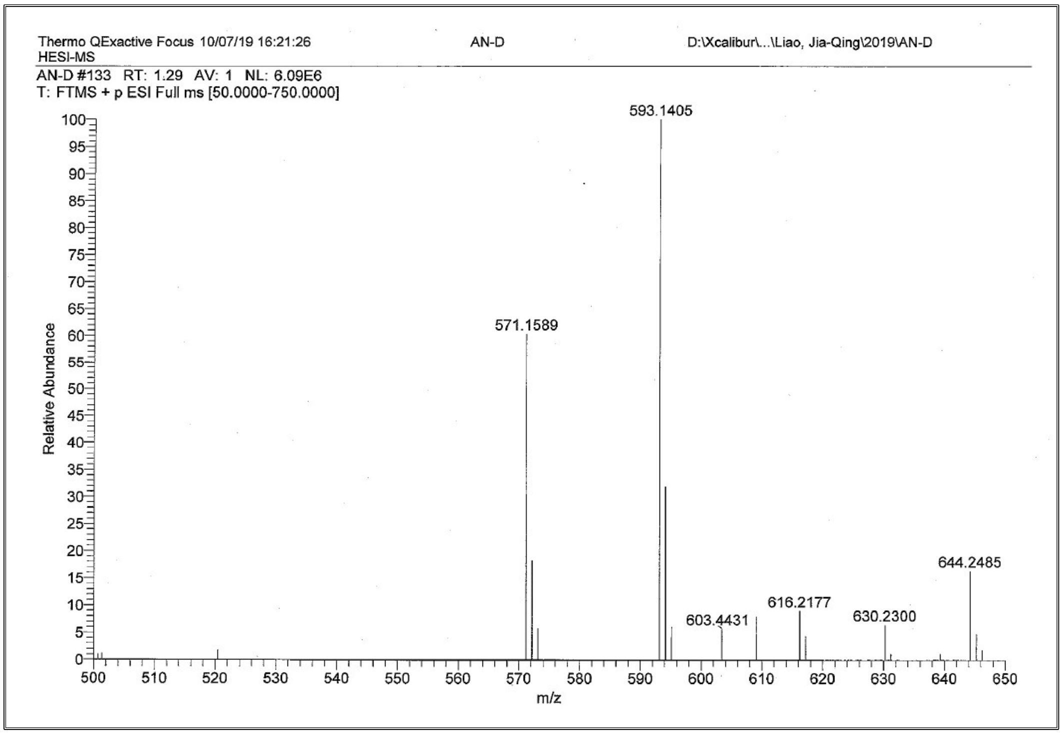

Figure S16 : ESI-Mass spectrum (positive ion mode) of compound 6.

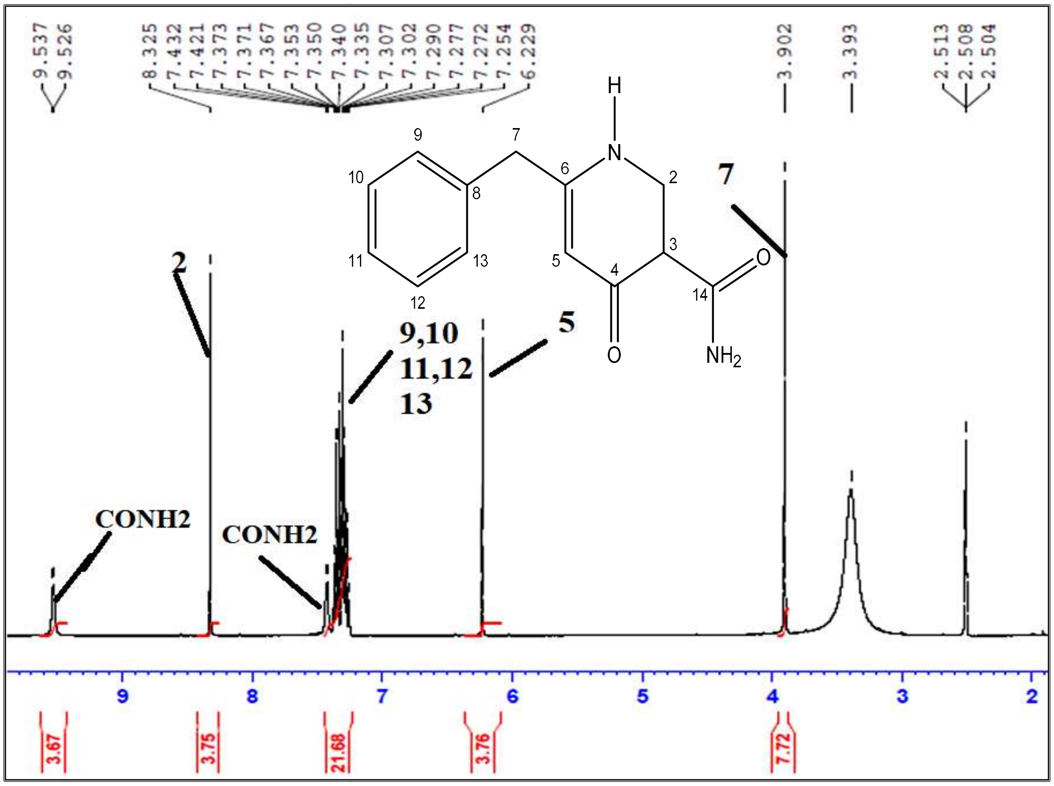

Figure S17 : ^{1}H-NMR spectrum (400 MHz, $CDCl_3$) of compound 7.

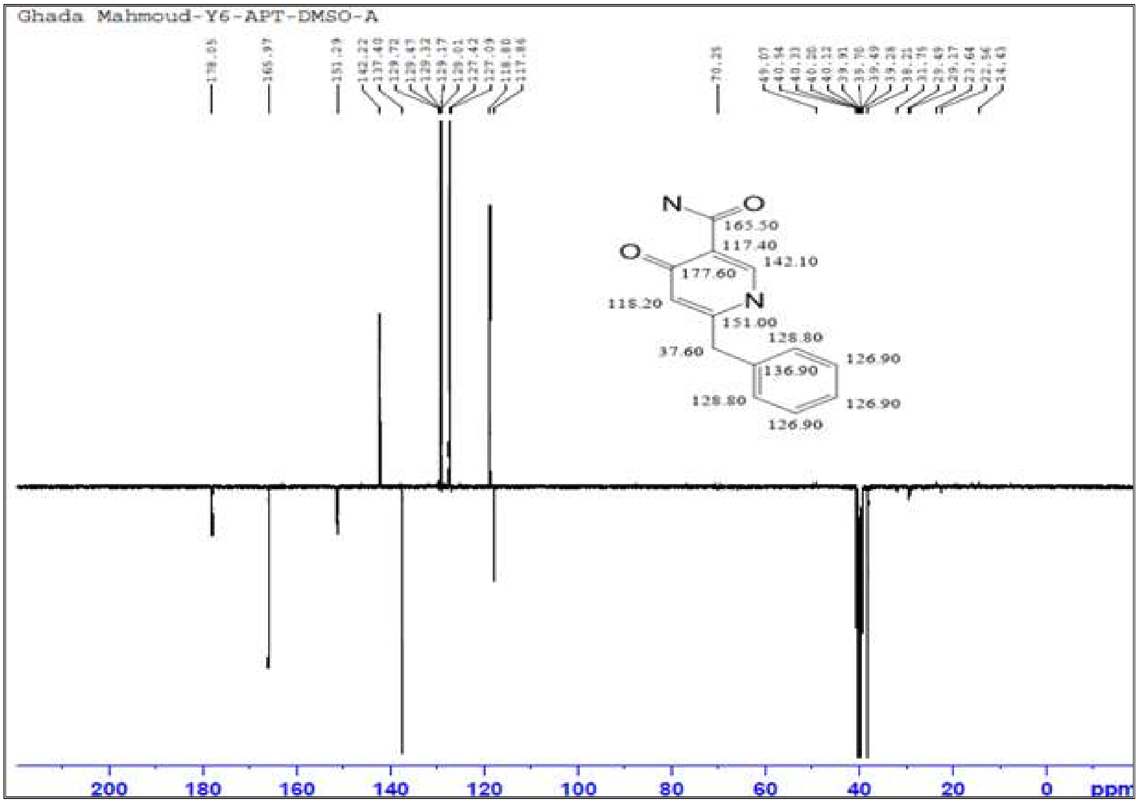

Figure S18 : APT spectrum (100 MHz, $CDCl_3$) of compound 7.

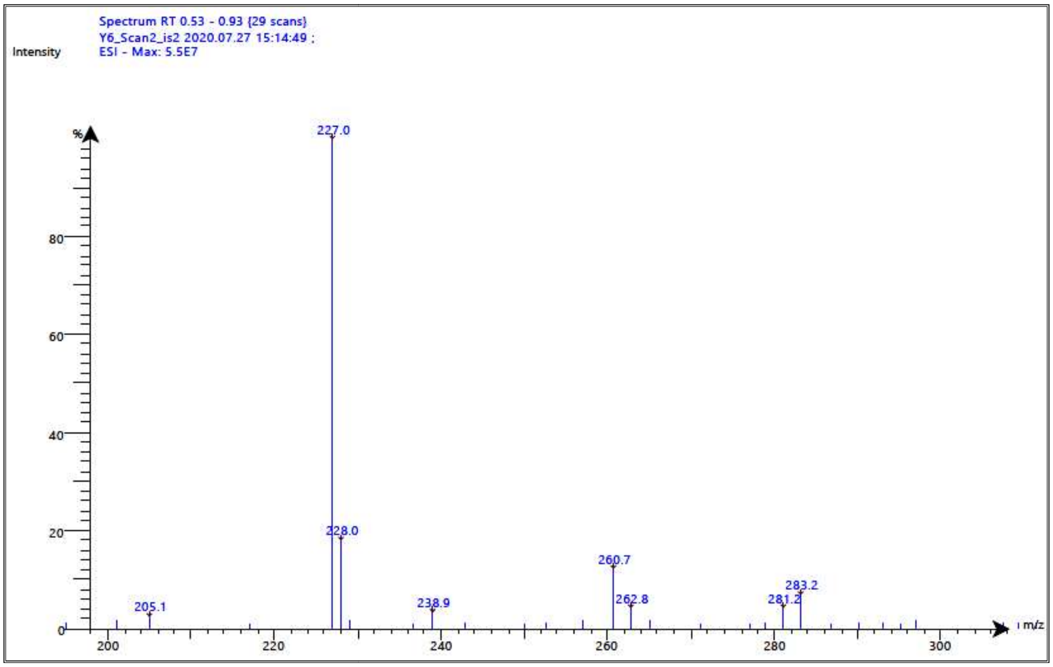

Figure S19 : ESI-Mass spectrum (negative ion mode) of compound 7.

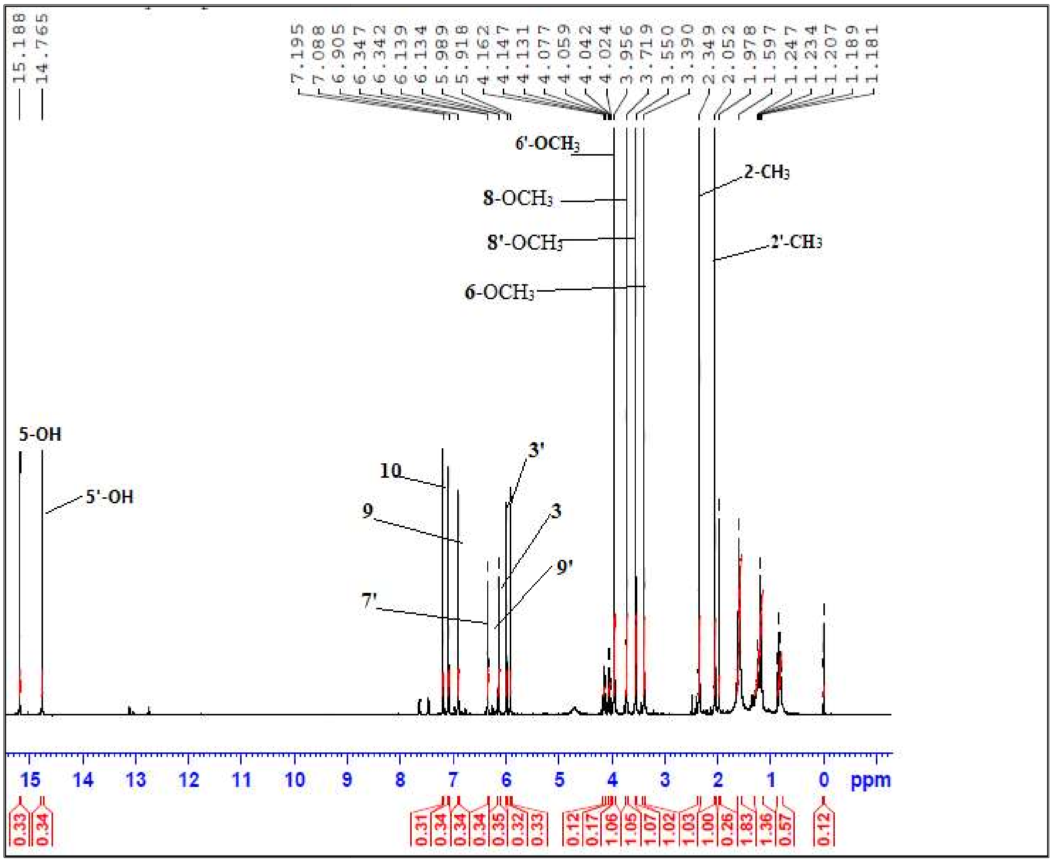

Figure S20 : ^{1}H-NMR spectrum (400 MHz, $CDCl_3$) of compound 3(additional to Figure S10).

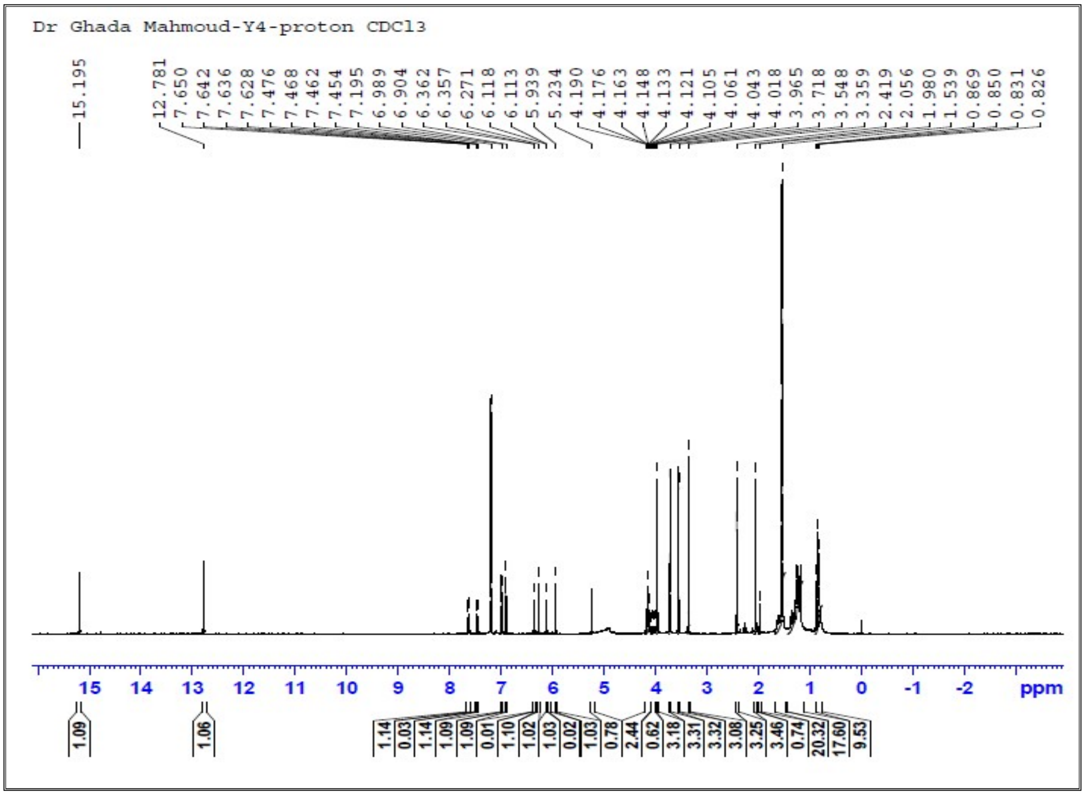

Figure S21 : ^{1}H-NMR spectrum (400 MHz, $CDCl_3$) of compound 4 (additional to Figure S13).

Table 1. Results of UV absorbance of compounds 3, 4 and 7 upon addition of $AgNO_3$ solution after 1 h. and after 48 h.

Absorbance at 450 nm	T/B*	Aurasperone A		Fonsecinone A		Aspernigrin A	
		Conc.	Absorbance	Conc.	Absorbance	Conc.	Absorbance
**after 1 h**	T<br>B	2.85 mg	0.301<br>0.128	2.85 mg	0.373<br>0.156	1.425 mg	0.144<br>0.072
	T<br>B	1.425 mg	0.15<br>0.094	1.425 mg	0.152<br>0.11	0.712 mg	0.651<br>0.066
	T<br>B	0.712 mg	0.165<br>0.047	0.712 mg	0.133<br>0.068	0.356 mg	0.139<br>0.062
	T<br>B	0.356 mg	0.164<br>0.047	0.356 mg	0.098<br>0.072	0.178 mg	0.574<br>0.082
	T<br>B	0.178 mg	0.119<br>0.041	0.178 mg	0.162<br>0.087	0.089 mg	0.142<br>0.085
	T<br>B	0.089 mg	0.11<br>0.041	0.089 mg	0.125<br>0.078	0.044 mg	0.154<br>0.084
**after 48 h**	T<br>B	2.85 mg	1.098<br>0.122	2.85 mg	0.523<br>0.138	1.425 mg	1.119<br>0.065
	T<br>B	1.425 mg	0.851<br>0.093	1.425 mg	0.714<br>0.102	0.712 mg	0.962<br>0.06
	T<br>B	0.712 mg	0.91<br>0.043	0.712 mg	0.424<br>0.066	0.356 mg	0.208<br>0.06
	T<br>B	0.356 mg	0.9<br>0.04	0.356 mg	0.286<br>0.07	0.178 mg	0.574<br>0.074
	T<br>B	0.178 mg	0.314<br>0.04	0.178 mg	0.517<br>0.084	0.089 mg	0.268<br>0.077
	T<br>B	0.089 mg	0.302<br>0.039	0.089 mg	0.24<br>0.079	0.044 mg	0.554<br>0.076

T*: Test **/ B*:** Blank
