# Supplementary material for: Acetylcholine esterase inhibitory activity of green synthesized nanosilver by naphthopyrones isolated from marine-derived Aspergillus niger
Source: PLoS One. 2021 Sep 10;16(9):e0257071. doi: 10.1371/journal.pone.0257071 (PMC8432876; doi:10.1371/journal.pone.0257071)
Supplement: Attachment — Submitted filename: Response to Reviewers.docx [file pone.0257071.s002.docx]

**Journal Requirements:**

1- Some flaws were corrected in the manuscript to meet PLOS ONE's style requirements.
2- Regarding the field work, the marine material was collected by Prof. Dr/ Tarek Abdelaziz and Dr/ Mohammed El-Metwally at the National Institute of Oceanography and Fisheries, Red Sea Branch, Hurghada, Egypt (the 1^st^ one is a co-author). The samples were collected from the coral reef in front of the institute in Hurghada. The researchers of the institute are allowed to carry out their experiments, including collecting samples with no official permit. Furthermore, this organism was recently listed among the invasive species of the Mediterranean Sea  Two organisms only were collected and their fresh weight was less than 100 gm.
3- The answer was added to Methods section in the manuscript. The samples were collected from the coral reef in front of the National Institute of Oceanography and Fisheries, Red Sea Branch, Hurghada, Egypt by SCUBA diving at depth (5 m), (located at 27˚17’04.8”N, 33˚46’30.3”E).

**Reviewer #1:**.

1- **(a)** X-ray diffraction (XRD) analysis is used to check the crystalline nature of silver nanoparticles. So, it should be done on the dried powder. In our case, after lyophilization it was a sticky material at the end of the wasserman test tube (as seen in Figure 2) because the amount of starting compound entering the synthesis was very little so, it cannot be in a powder form or form crystals. In addition, other published papers performed (XRD) analysis because they used total extract in green synthesis of silver nanoparticles. Consequently, they have large amount but in our experiment we used pure isolated compounds with little yield.

**(b)** XPS is used for elemental analysis using solid surface and Raman is used for chemical structure identification and detection of contamination and impurities and give information about phase and polymorphism. In case of silver nanoparticles synthesized by total extract, Raman and XPS were performed as the total extract may contain different organic compounds and some elements as impurities but here we used pure organic compounds which were isolated by pure organic solvents and their NMR data were attached in the supplementary file. Therefore, probability of the presence of other elements is almost non-existent. Also these analyses are too expensive in our country due to low facilities and lack of funding. There is only one equipment and it is reserved for several months.

2- Regarding the mechanism interpretation and analysis to support the conclusion, the effect of silver nanoparticles alone on acetylcholine esterase enzyme was performed and its IC_50_ is 2.29 ± 0.013 uM/ml. This confirms that the pure isolated organic compounds have synergistic effect on acetylcholine esterase inhibition after capping with silver nanoparticles possibly by stabilizing them for a longer time to exert its effect.
3- The quality of all figures was dimproved and resolution was adjusted between 300-600 dpi to meet PLOS ONE's style figure requirements.

**Reviewer #2:**

1- The whole manuscript was screened and some were rewritten with proper English.
2- Abstract was rewritten defining the novelty of work with some quantitative data.
3- The first paragraph was revised with a full formatting.
4- In paragraph 3, the hypothesis and the novelty of the work were added.
5- Some recent papers of your suggestions were added.
6- Section 2.3 was added to section 2.2.
7- Section 2.5 was combined with section 2.4 under one subheading.
8- Proper annotations were used as possible as we can.
9- Some details were added Methods section.
10- The compound number was replaced by the full name of the compound in the result section.
11- Table 1 presented as a graph in Figure 4. The SPR was between 441 to 463 nm and was added to the manuscript.
12- Figure 2 was redrawn. The resolution was adjusted between 300-600 dpi to meet PLOS ONE's style figure requirements.
13- Table 2 became Table 1 and was revised.
14- Figure 4 was redrawn. The resolution was adjusted between 300-600 dpi to meet PLOS ONE's style figure requirements.
15- Discussion was revised, clarified and significance of the findings was discussed in section 3.3
16- Recent papers were cited.
