# Supplementary material for: Acetylcholine esterase inhibitory activity of green synthesized nanosilver by naphthopyrones isolated from marine-derived Aspergillus niger
Source: PLoS One. 2021 Sep 10;16(9):e0257071. doi: 10.1371/journal.pone.0257071 (PMC8432876; doi:10.1371/journal.pone.0257071)
Supplement: Attachment — Submitted filename: Response to reviewers.docx [file pone.0257071.s003.docx]

**Response to reviewers**

**1-** Adressed to reviewer #2. (Previous round).

**2-** Adressed to reviewer #2. (Technically sound).

**3-** Adressed to reviewer #2. (Statistical analysis).

**4-** Adressed to reviewer #2. (Data availability).

**5-** Adressed to reviewer #2. (Standard English).

**6-** The reviewer suggested a more focus on discussion part and separate it from results which were actullay did. The results (P.6) and discussion (P.9) section was separated and the results were described in how and why pattern. Really, it is a crucial part. Thank you for your opinion and effort.

**7-** This item also was addressed to reviewer #2.

We did our maximum efforts to improve the manuscript guided by editor and reviewers' comments and we hope that the final version will be satisfactory for respected editor and reviewers.

Thank you
